# Incidence of Venous Thromboembolism After Surgery for Acute Stanford Type A Aortic Dissection

**DOI:** 10.7759/cureus.103608

**Published:** 2026-02-14

**Authors:** Masato Saitoh, Takuma Yamasaki, Tomoaki Tanabe, Shuichi Tochigi, Daiki Hirayama, Kiyotaka Aoyama, Imun Tei

**Affiliations:** 1 Department of Cardiothoracic Surgery, Ayase Heart Hospital, Adachi, JPN

**Keywords:** acute aortic dissection, cardiovascular surgery, deep vein thrombosis, pulmonary thromboembolism, venous thromboembolism

## Abstract

Background

The incidence of postoperative deep vein thrombosis (DVT) and pulmonary thromboembolism (PTE) following acute Stanford type A aortic dissection (AADA) has not been investigated. Therefore, this study aimed to determine the incidence and risk factors of DVT and PTE following AADA.

Methods

This study included all patients who underwent emergency surgery for AADA at Ayase Heart Hospital, Tokyo, Japan, between April 1, 2023, and October 31, 2025. We excluded patients with preoperative venous thromboembolism (VTE), those who could not undergo postoperative lower extremity venous ultrasonography, and those who were undergoing anticoagulant therapy at the time of the postoperative ultrasound examination. DVT was assessed using lower extremity venous ultrasonography immediately before surgery and on the second postoperative day. Patients with DVT on postoperative lower extremity venous ultrasonography were grouped into the DVT group, while those who did not have DVT were grouped into the non-DVT group. The primary endpoint was the incidence of DVT, and the secondary endpoint was the incidence of PTE.

Results

Ninety-three of the 115 cases were included in the analysis. The DVT and non-DVT groups comprised 15 (16.1%) and 78 patients (83.9%), respectively. The incidence of PTE was significantly higher in the DVT group than in the non-DVT group (two cases: 13.3% vs. 0 cases: 0%; p = 0.01; χ^2^ = 10.63; effect size: 0.34). No significant differences were found between the two groups in terms of other intraoperative and postoperative outcomes. The low incidence of DVT events resulted in insufficient statistical power, precluding the identification of independent risk factors in the multivariate analysis.

Conclusions

AADA often presents with elevated preoperative D-dimer levels, which frequently persist into the postoperative period. Consequently, the D-dimer level may not be a reliable indicator of VTE in these patients. The incidence of PTE in patients with AADA was 2.2% (n=2/93), the critical importance of both prevention and early detection of DVT. Lower extremity venous ultrasonography may be effective for DVT screening in patients after AADA surgery, complementary to conventional DVT prevention protocols.

## Introduction

Pulmonary thromboembolism (PTE) is a serious perioperative complication, and its main cause is deep vein thrombosis (DVT).

After cardiovascular surgery, patients often receive anticoagulant therapy, and a low incidence of DVT has been reported [1,2]. The incidence of DVT after cardiovascular surgery in our previous study was 7.3%, with no PTE noted [3]. Previous studies have reported the incidence of PTE after coronary artery and aortic surgery [4-9].

Postoperative antithrombotic therapy is frequently administered, particularly for valvular disease and coronary artery disease, to prevent thrombus formation on prosthetic valves and bypass graft occlusion. From the perspective of deep DVT prophylaxis, these treatments are considered highly effective. Conversely, as postoperative antithrombotic therapy is less frequently administered following major vascular surgery, these patients may be at a higher risk for the incidence of DVT compared to those undergoing other cardiovascular diseases.

In particular, in the acute postoperative phase of aortic dissection, enhanced coagulation-fibrinolysis leads to a hypercoagulable state, predisposing patients to thrombus formation. However, treatment is often not administered to control postoperative bleeding. Furthermore, surgeries for aortic dissection frequently require blood transfusions to manage perioperative bleeding. We propose that coagulation factors present in fresh frozen plasma (FFP) potentiate this RBC-microparticle interaction and increase the likelihood of DVT [10]. Furthermore, they report that RBC transfusion is associated with increased risk of DVT after cardiac surgery in a dose-dependent fashion that is exacerbated when accompanied by FFP [10]. Our prior research similarly identified blood transfusion as a significant risk factor for postoperative DVT in patients undergoing cardiovascular procedures [3]. Therefore, the perioperative period of acute aortic dissection can be considered the highest risk phase for the incidence of DVT within the field of cardiovascular surgery. The association with blood transfusion was considered particularly important. However, the incidence of postoperative DVT and PTE after acute AADA has not been determined, and their frequencies remain unknown [11]. Therefore, this study aimed to clarify the incidence of DVT on the second postoperative day and acute phase PTE in patients undergoing surgery for acute AADA while investigating risk factors such as blood transfusion.

## Materials and methods

Study design, duration, and ethical considerations

This observational study was conducted at Ayase Heart Hospital, Tokyo, Japan, between April 1, 2023, and October 31, 2025, and was approved by the Ayase Heart Hospital Research Ethics Committee (approval no.: 2023-0001-1). Opt-out was implemented on the hospital website to ensure patients' right to refuse. The requirement for patient consent was waived considering the emergency of the patients' conditions. This study was conducted in accordance with the Declaration of Helsinki and the Ethical Guidelines for Medical and Health Research Involving Human Subjects.

Sample size determination

Saitoh et al. reported a 7.3% incidence rate of postoperative DVT [3]. Given that the incidence of DVT in patients with acute aortic dissection was expected to be higher than previously reported, the sample size was calculated assuming a DVT incidence of 15%. With a statistical power of 0.80 and a two-sided alpha level of 0.05, the required sample size was determined using SPSS Statistics version 30 (IBM Japan, Ltd., Tokyo, Japan). The required sample size was determined to be 111 cases. Factoring in an expected dropout rate of approximately 10%, the final sample size was set at 120 cases.

Study population

This study included all patients who underwent surgery for acute Stanford type A aortic dissection (AADA) at our hospital during the study period. Thus, 115 individuals were included in the study. We excluded patients with intraoperative death, a history of PTE, or preoperative DVT confirmed by ultrasonography. Furthermore, those who died or were transferred before the second postoperative day and could not undergo lower extremity venous ultrasonography were excluded. While anticoagulation is effective for preventing thrombus formation, it was withheld in the postoperative period following aortic dissection surgery due to concerns regarding hemorrhagic complications. To determine the true incidence of DVT, we excluded patients on anticoagulation during their first postoperative ultrasound, thereby eliminating the confounding effect of such therapy. Those who presented with DVT on postoperative lower extremity venous ultrasonography were grouped as the DVT group, while those without this modality were grouped as the non-DVT group. Data on patient demographics, life history, medical history, surgical procedure, blood tests, postoperative complications, and postoperative course were collected from the electronic charts.

Endpoints

The primary and secondary endpoints in this study were the incidences of DVT and PTE, respectively.

Lower extremity venous ultrasonography

To minimize observer bias, lower extremity venous ultrasonography was performed and evaluated by at least two highly experienced, certified sonographers at our hospital. DVT was assessed using lower extremity venous ultrasonography immediately before surgery and on the second postoperative day. In our hospital, walking is initiated on the third postoperative day, and therefore, lower extremity venous ultrasonography was performed on the second postoperative day. On the first postoperative day, hemodynamic and respiratory stability were prioritized; therefore, lower extremity venous ultrasonography was performed only when symptoms suggestive of DVT were observed. The imaging protocol was as follows. The thrombus was evaluated using the compression technique. If a thrombus was suspected, the color Doppler method was performed to confirm the blood flow deficit. The search area extended from the common to the inferior femoral vein. A thrombus located more centrally than the popliteal vein was defined as the central type. A thrombus located peripheral to the popliteal vein, including the anterior tibial, posterior tibial, peroneal, gastrocnemius, and soleal veins, was defined as the peripheral type. Diagnosis of DVT was primarily performed using lower extremity venous ultrasonography. Contrast-enhanced computed tomography (CT) was considered only when ultrasonography provided insufficient evaluation.

Computer tomography

In accordance with our institutional postoperative protocol, contrast-enhanced CT scans of the cervical to the femoral region were conducted on the seventh postoperative day after surgery for aortic dissection. In exceptional cases where respiratory deterioration suggested pulmonary thromboembolism, contrast-enhanced CT was performed immediately under the attending physician's discretion. The CT scans were evaluated by multiple cardiovascular surgeons, as there was no staff diagnostic radiologist at our hospital. To minimize observer bias, CT-based diagnosis of PTE was independently reviewed by at least two board-certified cardiovascular surgeons. PTE was diagnosed based on the identification of pulmonary arterial thrombi using contrast-enhanced CT.

DVT prophylaxis and treatment

Postoperative compression therapy was initiated immediately following surgery in accordance with the standard prevention protocol of our hospital. Patients with preoperative peripheral malperfusion and those who underwent great saphenous vein harvesting did not undergo ipsilateral compression therapy. This therapy was also not administered in cases in which patients could be weaned from mechanical ventilation within 12 hours postoperatively, and early ambulation was expected. Compression therapy was continued until discharge was finalized.

Prophylactic anticoagulation for DVT was fundamentally avoided due to concerns regarding postoperative hemorrhage and false lumen patency. However, for patients who underwent concomitant valve replacement, warfarin was initiated on the first postoperative day in accordance with our institutional protocol. In principle, antithrombotic therapy was not administered for peripheral DVT.

Statistical analyses

The distribution of continuous variables was assessed for normality using the Shapiro-Wilk test prior to applying appropriate inferential tests. Continuous variables are presented as mean ± standard deviation and were analyzed using Student's t-test or Mann-Whitney U test. Categorical variables are presented as numbers (%) and were analyzed using the chi-squared test for between-group comparisons. Multivariable analysis was performed using binary logistic regression. The response variable was the incidence of postoperative DVT. The explanatory variable was the total intraoperative blood transfusion volume, cardiopulmonary bypass time, duration of postoperative ventilator use, and number of days until ambulation based on the report by Saitoh et al. [3]. Multicollinearity was assessed using the variance inflation factor (VIF), with a VIF value of ≥5 indicating the presence of multicollinearity. Missing data were minimal and were handled using complete-case analysis without imputation. Statistical significance was defined as p < 0.05. For each variable, effect sizes were calculated to indicate the substantive significance of the relationships. Cramer's V was used for categorical variables, and Cohen's d was used for continuous variables. Effect sizes were categorized as small (<0.3 for Cramer's V, <0.5 for Cohen's d), medium (0.3-0.5 for Cramer's V, 0.5-0.8 for Cohen's d), or large (≥0.5 for Cramer's V, ≥0.8 for Cohen's d) [12]. The t-statistic for t-tests, the U-statistic for Mann-Whitney U tests, and the chi-square statistic for chi-square tests were computed as the test statistics. Statistical analyses were performed using SPSS Statistics version 30 (IBM Japan, Ltd., Tokyo, Japan).

## Results

No cases of preoperative DVT were observed. Additionally, as all patients were successfully evaluated using lower limb venous ultrasound, none required CT imaging for further assessment. After excluding one case of preoperative PTE, one case of immediate postoperative death, one case of transfer due to postoperative cerebral infarction, and 19 patients with anticoagulant therapy by the second postoperative day, 93 of 115 eligible cases were analyzed. The planned sample size was not achieved during the study period. DVT was observed in 15 of 93 cases (16.1%). DVT lesions were identified as follows: 11 patients had one DVT, four patients had two DVTs, and all DVTs were peripheral. As all patients had peripheral DVT, they did not receive additional anticoagulation for DVT. The use rate of compression stockings was 33.3% (5/15) and 47.4% (37/78) in the DVT and non-DVT groups, respectively. Preoperative malperfusion in two of the 15 cases in the DVT group, both of which were cerebral malperfusion. There were no significant differences in preoperative age, sex, EuroSCORE, or past medical history. Preoperative D-dimer levels were significantly higher in the non-DVT group (Tables 1-2).

**Table 1:** Patient characteristics and preoperative state DVT, deep venous thrombosis; LVEF, left ventricular ejection fraction Values are presented as mean ± SD or n(%) Test statistics were reported as t-statistic (T) for t-tests and chi-square statistic (χ^2^) for chi-square tests.

Variables	DVT(＋) (n = 15)	DVT(ｰ) (n = 78)	Test statistic	Effect size	P-value
Age (years)	70.3 ± 14.9	67.5 ± 14.5	T=0.69	0.19	0.49
Female	10 (66.7)	39 (50.0)	χ^2^=1.4	0.12	0.24
Body mass index	25.9 ± 6.1	24.9 ± 5.5	T=0.64	0.18	0.52
Euro score	12.0 ± 3.8	11.3 ± 3.7	T=0.72	0.2	0.48
LVEF (%)	59.7 ± 8.5	61.7 ± 7.5	T=0.92	0.26	0.36
False lumen patency on CT					
Patent	5 (35.7)	39 (50.6)	χ^2^=7.01	0.28	0.07
Patial patency	2 (14.3)	19 (24.7)			
Ulcer-like projection	4 (28.6)	5 (6.5)			
Malperfusion	2 (13.3)	24 (30.8)	χ^2^=1.9	0.14	0.17
Medical history					
Hypertension	12 (80.0)	65 (83.3)	χ^2^=0.1	0.03	0.75
Diabetes	2 (13.3)	4 (5.1)	χ^2^=1.4	0.12	0.24
Dyslipidemia	4 (26.7)	29 (37.2)	χ^2^=0.61	0.08	0.44
Heart failure	0	4 (5.1)	χ^2^=0.8	0.09	0.37
Cerebrovascular disease	0	6 (7.7)	χ^2^=1.23	0.12	0.27
Chronic renal dysfunction (eGFR < 45)	6 (40.0)	23 (29.5)	χ^2^=0.65	0.08	0.42
Cancer	0	5 (6.4)	χ^2^=1.02	0.1	0.31
Antithrombotic drug	0	13 (16.7)	χ^2^=2.91	0.18	0.09
Smoking history	7 (46.7)	41 (52.6)	χ^2^=0.18	0.04	0.68

**Table 2:** Laboratory data DVT, deep venous thrombosis; PT-INR, international normalized ratio of prothrombin time; APTT, activated partial thromboplastin time; BUN, blood urea nitrogen; CRP, C-reactive protein; HbA1c, hemoglobin A1c; BNP, brain natriuretic peptide Values are presented as mean ± SD. Test statistics were reported as t-statistic (T) for t-tests.

Variables	DVT(＋) (n = 15)	DVT(ｰ) (n = 78)	Test statistic	Effect size	P-value
Preoperative laboratory data					
PT-INR	1.1±0.2	1.1±0.3	T=0.23	0.06	0.82
APTT (s)	31.6±4.9	32.0±7.4	T=0.18	0.05	0.86
D-dimer (µg/mL)	31.5±34.9	43.4±55.8	T=0.8	0.22	0.43
Hematocrit (%)	37.2±5.7	38.5±7.5	T=0.63	0.18	0.53
BUN (mg/dL)	27.2±21.1	20.7±10.6	T=1.81	0.51	0.07
Creatinine (mg/dL)	1.8±1.8	1.3±1.3	T=1.31	0.37	0.19
BNP (pg/mL)	322.5±878.9	93.9±159.5	T=2.13	0.6	0.04
Postoperative 7-day laboratory data					
PT-INR	1.4±0.2	1.4±0.3	T=0.08	0.02	0.94
APTT (s)	46.5±22.6	43.1±12.9	T=0.82	0.23	0.42
D-dimer (µg/mL)	20.7±15.7	34.2±44.5	T=1.16	0.33	0.25
Hematocrit (%)	38.1±4.9	36.7±4.8	T=1.03	0.29	0.3
BUN (mg/dL)	49.9±22.0	47.6±17.9	T=0.45	0.13	0.66
Creatinine (mg/dL)	2.3±2.1	2.1±1.7	T=0.37	0.1	0.72

There were no significant differences between the two groups in terms of operative time, cardiopulmonary bypass time, or total intraoperative blood transfusion volume (Table 3).

**Table 3:** Operative procedure-related data DVT, deep venous thrombosis; HAR, hemi arch replacement; TAR, total arch replacement; PAR, partial arch replacement; CPB, cardiopulmonary bypass; OP, operation Values are presented as mean ± SD or n(%). Test statistics were reported as t-statistic (T) for t-tests and chi-square statistic (χ^2^) for chi-square tests.

Variables	DVT(＋) (n = 15)	DVT(ｰ) (n = 78)	Test statistic	Effect size	P-value
Operative procedure					
HAR	9 (60.0)	39 (50.0)	χ^2^=1.16	0.11	0.56
TAR	4 (26.7)	32 (41.0)			
PAR	2 (13.3)	7 (9.0)			
Re-operation	0	2 (2.6)	χ^2^=0.39	0.07	0.53
Procedure-related data					
Anesthesia time (min)	322.2 ± 56.6	353.1 ± 77.6	T=1.47	0.41	0.15
OP time (min)	270.8 ± 55.4	303.4 ± 74.0	T=1.62	0.46	0.11
CPB time (min)	156.9 ± 47.3	162.1 ± 47.6	T=0.39	0.11	0.7
Total blood products (IU)	67.4 ± 9.7	70.5 ± 16.0	T=0.73	0.2	0.47
Red blood cell (IU)	16.9 ± 4.9	17.3 ± 6.3	T=0.24	0.07	0.81
Fresh frozen plasma (IU)	15.2 ± 2.6	18.4 ± 6.3	T=1.91	0.54	0.06
Platelet (IU)	35.3 ± 5.1	34.9 ± 7.7	T=0.22	0.06	0.82
Heparin dose (mL)	18.8 ± 5.6	18.8 ± 4.4	T=0.03	0.01	0.97
Protamine dose (mL)	24.5 ± 9.8	28.20 ± 8.8	T=1.46	0.41	0.15

There were no significant differences in the operative outcomes between the two groups (Table 4).

**Table 4:** Postoperative outcomes and complications DVT, deep venous thrombosis; ICU, intensive care unit; POD, postoperative day; NOAC, novel oral anticoagulants; DOAC, direct oral anticoagulants; SAPT, single antiplatelet therapy; DAPT, dual antiplatelet therapy; PTE, pulmonary thromboembolism; CVD, cerebrovascular disease; CRRT, continuous renal replacement therapy Values are presented as mean ± SD or n(%). Test statistics were reported as t-statistic (T) for t-tests and chi-square statistic (χ^2^) for chi-square tests.

Variables	DVT(＋) (n = 15)	DVT(ｰ) (n = 78)	Test statistic	Effect size	P-value
Hospital stay (day)	25.5 ± 12.8	24.6 ± 13.1	T=0.25	0.07	0.81
ICU stay (POD)	11.1 ± 10.4	8.7 ± 6.9	T=0.14	0.31	0.27
Ventilator time (h)	64.6 ± 86.2	53.9 ± 78.1	T=0.48	0.14	0.63
Drainage for 24 hours (mL)	834.4 ± 606.6	984.3 ± 601.7	T=0.88	0.25	0.38
Passive exercise of the lower limb (POD)	1.7 ± 1.1	1.4 ± 1.3	T=0.95	0.27	0.34
50-m walking (POD)	5.4 ± 2.3	7.4 ± 6.7	T=0.98	0.32	0.33
Oral intake (POD)	3.0 ± 1.4	3.1 ± 3.4	T=0.09	0.03	0.93
CVC decannulate (POD)	4.2 ± 1.8	3.2 ± 2.5	T=1.39	0.39	0.17
Postoperative patent false lumen					
Complete thrombosed	8 (66.7)	30 (40.0)	χ^2^=2.99	0.19	0.22
Partially thrombosed	3 (25.0)	34 (45.3)			
Complication					
Re-thoracotomy	1 (6.7)	5 (6.4)	χ^2^=0.001	0.01	0.97
PTE	2 (13.3)	0	χ^2^=10.63	0.34	0.01
Prolonged ventilation (>72 h)	3 (20.0)	17 (21.8)	χ^2^=0.02	0.02	0.88
Re-intubation	2 (13.3)	4 (5.1)	χ^2^=1.4	0.12	0.24
Pneumonia	0	6 (7.7)	χ^2^=1.23	0.12	0.27
CVD	5 (33.3)	18 (23.1)	χ^2^=0.71	0.09	0.4
Paraplegia	0	1 (1.3)	χ^2^=0.19	0.05	0.66
CRRT	1 (6.7)	11 (14.1)	χ^2^=0.62	0.08	0.43
Delirium	2 (13.3)	21 (26.9)	χ^2^=1.25	0.12	0.26

The incidence of PTE among postoperative complications was 2/93 cases (2.2%). The DVT group had a significantly higher incidence of PTE and paraplegia (Table 4). Multivariate analysis did not reveal any identifiable risk factors (Table 5). A post-hoc power analysis revealed a power of 0.05. Due to the limited number of DVT events (n=15), this study was underpowered to perform a robust multivariate analysis.

**Table 5:** Risk factors for postoperative DVT occurrence CPB: cardiopulmonary bypass

Variables	Odds ratio	95% Confidence interval	P value
Total blood products	0.94	0.94–1.05	0.79
CPB time	0.99	0.98–1.01	0.42
Ventilator time	1.01	0.99–1.03	0.41
50-m walking	0.86	0.65–1.15	0.32

## Discussion

This study investigated the incidence of newly developed DVT in patients undergoing surgery for AADA by performing lower extremity venous ultrasonography before and after surgery. Patients with DVT on postoperative lower extremity venous ultrasonography were grouped as the DVT group, while those without were grouped as the non-DVT group. We hypothesized that an increased total volume of intraoperative blood transfusion elevates the risk of postoperative DVT. The incidences of DVT and PTE were 16.1% (n = 15/93) and 2.2% (n = 2/93), respectively. AADA often presents with elevated preoperative D-dimer levels, which frequently persist into the postoperative period. Consequently, the D-dimer level may not be a reliable indicator of VTE in these patients. Lower extremity venous ultrasonography is considered the most useful method for detecting postoperative DVT following AADA.

Postoperative circulatory dynamics in acute aortic dissection are often unstable owing to factors such as bleeding. Moreover, blood pressure management is crucial for preventing paraplegia and maintaining tissue perfusion pressure in cases of malperfusion. Maintaining stable respiration is essential to prevent hypoxemia. Postoperative PTE can be fatal because of the collapse of the respiratory and circulatory systems. Therefore, the detection of DVT is crucial.

Reports indicate that thrombin concentration increases up to 24 hours postoperatively in acute aortic dissection [13]. Thrombin strongly promotes platelet aggregation, making it important for inhibiting postoperative bleeding. However, overactive thrombin generation may result in consumptive coagulopathy, which can trigger systemic thrombosis. As DVT contributes to postoperative PTE, early detection and intervention are critical. The D-dimer level is generally used as a biomarker of DVT. It has been reported that blood flow contact with the nonendothelialized false lumen results in the release of large amounts of cytokines, which activate the coagulation system and lead to the preoperative consumption of many clotting factors in patients with AADA [14]. Thus, it is highly probable that D-dimer levels are elevated preoperatively, and indeed, this study found high levels in both groups. Although entry resection is our standard surgical procedure, postoperative CT scans often reveal residual re-entry, and in this study, only 40.9% (n = 38/93) of cases achieved complete false lumen thrombosis after surgery. In such cases, the false lumen will remain in contact with the blood flow postoperatively, leading to significant consumption of clotting factors. D-dimer levels can be elevated not only preoperatively but also postoperatively. A prior study also reported elevated D-dimer levels postoperatively [15]. This suggests that D-dimer levels are often elevated after acute aortic dissection surgery, potentially masking thrombotic complications such as DVT and PTE, thus making it an unsuitable biomarker of VTE. Although all DVT cases in this study were peripheral, PTE was a complication in two cases. Postoperative PTE after cardiovascular surgery has a reported incidence of 0%-0.9% [1,8,16]. However, the incidence of postoperative PTE in acute aortic dissection in this study was 2.2%, which exceeds that noted in standard open-heart surgery. The incidence of postoperative DVT following surgery for acute aortic dissection was 16.1%, which suggestively higher than the 7.3%-13% reported for elective cardiovascular surgery [3,8]. However, as these figures were not statistically compared in the present study, further rigorous investigation is warranted. As previously mentioned, postoperative hypercoagulability and increased fibrinolysis are speculated to induce a systemic thrombotic state; however, this study did not yield results that definitively prove these mechanisms. Nevertheless, patients undergoing surgery for AADA are highly susceptible to postoperative DVT, a major cause of PTE. Although this study did not identify specific risk factors, the fact that AADA patients have a higher incidence of DVT compared to those undergoing other cardiac procedures suggests that comprehensive DVT prophylaxis should be implemented for all AADA cases. Furthermore, in addition to preventive measures, early detection of DVT remains critically important.

Given the insufficiency of the D-dimer level as a biomarker of DVT, lower extremity venous ultrasonography is the most reliable method for diagnosing postoperative DVT. However, it should be considered whether lower extremity venous ultrasonography should be performed in all cases after evaluating its cost-effectiveness. Recent reports have indicated the usefulness of P-selectin as a new biomarker of VTE [17]. However, no reports have investigated the changes in P-selectin levels after surgery for acute aortic dissection. Further validation is required to determine whether it is an appropriate biomarker of VTE complicated by acute aortic dissection. Regardless, given the diagnostic challenges of relying on a single biomarker for suspected VTE following acute aortic dissection surgery, it is imperative to confirm the diagnosis using multiple approaches in conjunction with imaging studies, such as lower extremity ultrasound and contrast-enhanced CT. Acute aortic dissection is characterized by dynamic perioperative changes in the coagulation and fibrinolysis system, resulting in a higher incidence of DVT compared to standard open-heart surgery. Furthermore, the annual incidence of acute aortic dissection is reported as 3.5 per 100,000 persons [18]. Because acute aortic dissection has a low incidence, the number of surgical procedures is correspondingly small. Therefore, performing lower limb ultrasonography in all cases would not impose a significant workload and cost burden, and the benefits of early VTE detection are considered to outweigh these costs.

In this study, the exclusion of patients on anticoagulation therapy at the first postoperative ultrasound allowed for the identification of the true DVT incidence, unconfounded by pharmacological intervention. Meanwhile, the incidence of DVT in patients receiving anticoagulant therapy has not been fully investigated. Given that the inclusion of this population could potentially alter the overall incidence rates of DVT following surgery for aortic dissection, further analysis is warranted. In general, anticoagulation is indicated for proximal DVT, whereas it is typically not indicated for isolated distal DVT. All DVT cases in this study were peripheral, and there was no indication for anticoagulation. However, because peripheral DVT can also be complicated by PTE, anticoagulation therapy should be considered in such cases. Although anticoagulation therapy after surgery for acute aortic dissection has not been associated with aortic-related death, it has been reported to increase the patency rate of the false lumen [19]. Anticoagulation may be necessary in cases of combined valve replacement or postoperative atrial fibrillation. However, careful consideration is required, considering the benefits of anticoagulation and the risks of bleeding complications and false lumen patency. The optimal anticoagulant agent for postoperative anticoagulation therapy in acute aortic dissection remains controversial. Nevertheless, considering the availability of antagonists and the ability to adjust their efficacy, warfarin or heparin are generally regarded as the safest anticoagulants to choose.

DVT prevention is essential to preclude PTE. Due to the specific nature of the disease, aggressive postoperative anticoagulation therapy is often contraindicated. Consequently, alternative preventive measures are essential. Excluding patients for whom lower limb compression is not feasible, such as those with peripheral ischemia and those who have undergone great saphenous vein harvesting - the most straightforward approach is the universal application of compression stockings or intermittent pneumatic compression. Furthermore, preventive interventions, such as the early initiation of passive lower-limb exercises and early mobilization, are important. The low implementation rate of postoperative lower extremity compression therapy in this study suggests that DVT incidence could have been further improved with adequate preventative measures. We believe that revising the standard DVT prophylaxis protocol to further increase the implementation rate of postoperative lower-limb compression therapy could reduce the incidence of postoperative VTE. Standard DVT biomarkers often lack predictive accuracy during the acute postoperative phase of acute aortic dissection. The variables analyzed in this study were conventional clinical parameters, none of which demonstrated significant predictive value. Given these findings, the clinical utility of novel biomarkers, such as P-selectin, warrants further investigation. In acute aortic dissection, the consumption of coagulation factors varies depending on the state of the false lumen, indicating that factors contributing to postoperative thrombus formation differ among individual cases. Although studies accounting for pre- and postoperative false lumen blood flow are desirable, the single-center design and insufficient sample size of this study precluded such analysis and hindered the identification of specific VTE risk factors. Therefore, studies with appropriate sample sizes are required to study false lumen blood flow and identify VTE risk factors.

Limitations

Given the single-center observational design and the failure to reach the target sample size, the possibility of inadequate statistical power cannot be ruled out. This was especially evident in the multivariate analysis, which may have been underpowered to identify definitive risk factors. Furthermore, there remains scope for improvement in the standardization of postoperative VTE prevention. Moreover, although we sought to minimize observer bias, the lack of a resident radiologist meant that potential bias in image interpretation could not be entirely excluded. Furthermore, as lower extremity venous ultrasonography was not performed on the first postoperative day, the precise onset of DVT during the hyperacute phase could not be identified. Additionally, the incidence of subacute DVT remains unknown because the study did not evaluate occurrences beyond the third postoperative day. Further investigation is warranted to address these points.

## Conclusions

The incidence of postoperative DVT was 16.1%, and that of PTE was 2.2% following surgery for AADA. D-dimer is an inadequate biomarker for the early detection of postoperative thrombotic complications in patients with AADA. Lower extremity venous ultrasonography is the most reliable method for the early detection of postoperative DVT in patients with AADA as of this moment. Since no risk factors for postoperative DVT were identified, adherence to standard DVT prophylactic measures and early detection remain crucial for the prevention of PTE.
